# Measuring the Monetary Value of Dental Implants for Denture Retention: A Willingness to Pay Approach

**DOI:** 10.2174/1874210601711010498

**Published:** 2017-09-14

**Authors:** Pedram Sendi, Nadine Bertschinger, Christina Brand, Carlo P. Marinello, Heiner C. Bucher, Michael M. Bornstein

**Affiliations:** 1 Basel Institute for Clinical Epidemiology and Biostatistics, Basel University Hospital, Basel, Switzerland; 2 Clinic for Reconstructive Dentistry and TMJ Disorders, University of Basel, Basel, Switzerland; 3 Applied Oral Sciences, Faculty of Dentistry, The University of Hong Kong, Prince Philip Dental Hospital, Sai Ying Pun, Hong Kong SAR, China

**Keywords:** Patient satisfaction, Quality of life, Willingness to pay (WTP), Willingness to accept (WTA), Dental implants, Denture retention

## Abstract

**Purpose:**

Two interforaminal dental implants in is a common treatment option for denture retention in edentulous patients. Economic methods to assess the patient’s quality of life include the willingness to pay (WTP) for implant treatment and willingness to accept (WTA) to forgo implant treatment. The purpose of this study was to assess the monetary value of implant retained complete dentures using WTP and WTA.

**Methods:**

We included a convenience sample of 16 patients from a previously published cohort study on the survival of immediately loaded implants in edentulous patients to assess WTP and WTA for this treatment option.

**Results:**

The average maximum WTP for implant treatment was 4606 (95% CI: 2991-6222) Swiss Francs. Out of the 16 patients, only 5 were willing to trade their implants for money, with a mean WTA of CHF 33'500 (range: 3000-100'000).****All patients would agree to undergo the implant surgery procedure again.

**Conclusion:**

The results of the present study show that most patients are not willing to trade the increase in quality of life after implant surgery against money, suggesting that WTA exceeds by large WTP for the same health condition.

## 1 INTRODUCTION

Two interforaminal dental implants in edentulous patients for denture retention is a common and cost-effective treatment option [1-5] It has been shown that this simple yet straightforward treatment modality substantially increases the quality of life of patients [6, 7] .

A number of studies have evaluated the increase in quality of life of edentulous patients when treated with implant-retained complete dentures [4-6, 8, 9]. Quality of life is usually assessed using either descriptive methods or methods that include health valuation [10]. Economic methods to estimate the monetary value of an intervention include willingness to pay (WTP) and the willingness to accept (WTA) approach [10-13].

Esfandiari *et al*. (2009) have evaluated the preferences of patients for implant overdentures in a Canadian setting using the WTP and WTA approach from the societal perspective [14]. However, costs and patient preferences for health states may vary in different settings and countries. Therefore, patient preferences for implant-retained overdentures in the edentulous lower jaw from a Swiss perspective using WTP and WTA were assessed. In addition, we adopted a patient's perspective that is more in line with the payment policy for dental care in Switzerland as dental care is usually not covered by the compulsory health insurance system.

## 2 MATERIALS AND METHODS

Patients were recruited from a previously published prospective cohort study including 20 edentulous patients who received 2 interforaminal dental implants in the lower jaw between December 2005 and July 2007 [1]. The inclusion and exclusion criteria were described in detail previously [1]. Out of the initial 20 patients, 16 could be enrolled in our study in 2013, 4 patients had either died or suffered from dementia and were unable to answer any questions in a meaningful way. We did not perform a sample size calculation as this would have entailed a randomized controlled trial controlling for all potential confounders.

Patients were sent letters to inform them about the planned study and then contacted by phone calls and asked whether they would be willing to participate. A consecutive phone interview of about 20 minutes duration was then conducted using a structured questionnaire. All questions were asked by the same trained interviewer. Since patient satisfaction in the original cohort study was assessed before and after implant placement using a visual analogue scale, the present study was also used to evaluate any change in valuation over time. The study was approved by the ethical committee for evaluating student studies at the University of Basel.

In the clinical cohort study assessing the survival of immediately loaded dental implants, patients had to pay a fixed amount of 1500 Swiss Francs (CHF) which corresponds to approximately a 50% discount of the real implant costs. The WTP/WTA exercise in the present study, however, was also designed to include the real costs of the treatment.

### 2.1 Questionnaire

In the first question, during follow-up (median time 5 years after implementation), patients were asked to rate the satisfaction with the current implant retained denture on an analogue scale between 0 (not satisfactory at all) and 10 (very satisfactory). In the second question patients were asked to rate *ex-post* the satisfaction with the complete denture before implant placement on a scale between 0 (not satisfactory at all) and 10 (very satisfactory). After answering question 2, patients were confronted with the value that they provided before implant placement and given the opportunity to correct that value. In the third question, patients were asked to rate *ex-post* the satisfaction with the complete denture 6 and 24 months after implant placement on a scale between 0 (not satisfactory at all) and 10 (very satisfactory). After answering question 3, patients were confronted again with the value that they actually provided 6 months or 24 months after implant placement, respectively, and given the opportunity to correct that value.

In the fourth question patients were asked whether they would be willing to undergo the implant placement procedure again. In the fifth question, patients were asked whether they would be willing to pay the reduced amount of CHF 1500 again to undergo the implant placement procedure to retain the prosthesis. If patients agreed, they were then asked in the sixth question whether they would also be willing to pay the full amount of CHF 3000 to undergo the implant placement procedure. If patients agreed to pay CHF 3000 to cover the implant placement procedure, the costs were increased in increments of 100 in question 7 until the maximum willingness to pay was achieved. If patients did not agree to pay CHF 3000, costs were reduced in increments of 100 until the maximum willingness to pay was achieved.

In the last question, patients were confronted with the hypothetical situation that they could be changed back in the same situation before implants were placed. They were then asked how much money they would need to be paid to accept that previous situation with a complete lower denture without implants.

### 2.2 Statistical Analysis

The data were first analyzed descriptively. Inter-group differences in VAS were compared using the non-parametric Wilcoxon signed-rank test. A *p*-value of 0.05 or less was considered as statistically significant. All analyses were conducted using Stata Version 11.2 (Stata Corp., College Station, Texas, USA).

## 3 RESULTS

Out of the 16 patients enrolled in our study, 50% were male / female, respectively. The average age was 73.5 years with a range between 58 and 87 years.

Five years after implant placement, the satisfaction score on a VAS (between 0 and 10) ranged from 8 to 10, with a mean of 9.25. After implant placement, patients rated their satisfaction with the previous unretained complete denture significantly lower (3.25 *vs* 5.43, *p*=0.0365, see Table **1**). The satisfaction score remained high 24 months after implant placement, even when given the opportunity to correct that value (Table **1**).

All patients would undergo the implant placement procedure again and all patients would agree again to pay the reduced fee of 1500 Swiss Francs that reflects a 50% discount rate. Out of the 14 patients, only 2 were not willing to pay the full amount of CHF 3000 that would have covered the actual costs. Out of the 16 patients, 15 were able to answer the WTP question (Table **2**). The average maximum WTP for implant placement was 4606 (95% CI: 2991-6222) Swiss Francs, with a range between CHF 1500 and 12'500. Out of the 16 patients, only 5 patients were willing to trade their implants against money in the WTA question, 11 patients were not willing to trade their implants against money at all (Table **2**).

## 4 DISCUSSION

In the present study it was shown that patient satisfaction after implant placement for lower denture retention in edentulous patients remains consistently high even after 5 years. Furthermore, the study shows that most patients are willing to pay more for implant treatment that the actual costs of the interventon. Finally, it was documented that most patients who received implant-retained are not willing to forgo the increase in quality of life even for high amounts of money.

Although there are a number of studies in the dental literature that document a substantial increase in quality of life after implant treatment to retain a lower denture, there is a paucity of data that use WTP and WTA methods to value dental interventions [7, 14] . In a similar study, Esfandiari and co-workers (2009) showed that 70% of the patients were willing to pay three times more than the actual costs to retain a complete lower denture with implants [14]. Our results are furthermore also in line with those from Esfandiari *et al*. (2009) who showed that the majority of patients (92%) wearing implant overdentures in the lower jaw were not willing to go back to their original state at any price [14]. Of note, all patients who were included in our study would undergo the implant surgery again, indicating that the benefit of implant retained overdentures outweighs the disutility associated with the surgical intervention.

It has been argued in the medical and economic literature, that WTP and WTA yield similar results, with WTA values usually being somewhat higher than WTP values [15] . The large difference between WTP and WTA values in our study, however, with WTA values reaching infinity in the majority of patients (*i.e*. patients not willing to trade implants for money), indicates that ability to pay may substantially influence the patient's willingness to pay for an intervention. Our results are also in line with widespread opinion that health is regarded as the highest good of mankind [16] .

This study has several limitations. First, we used a small convenience sample of 16 patients who received implant treatment to retain a mandibular complete denture. Therefore, the sample size is inadequate for more extensive inferential analyses. However, the descriptive results of the WTP/WTA exercise clearly document the benefit of implant retained overdentures and their impact on quality of life. Second, we conducted the study from the patient's perspective. This perspective may not be adequate for decisions at the societal level. However, since in Switzerland dental treatments are completely paid with out-of-pocket money, the results are consistent with the payment policy in Switzerland. Third, we included patients in our study who only had to pay 50% of the actual price in the original study, suggesting a lower socioeconomic status in most patients. Our results are therefore only transferable to a similar patient population.

## CONCLUSION

In conclusion, within the limitations of this study, the results suggest that in edentulous patients, the increase in quality of life by implant retained overdentures as compared to conventional dentures is substantial that patients are not willing to forgo at any price.

## Figures and Tables

**Table 1:** Patient satisfaction with implant treatment on a Visual Analogue Scale (VAS) over 24 months (n=16).

Patient satisfaction (0-10)	VAS (mean)	VAS (95% CI)	*p*-value 1 *vs* 2	*p*-value 1 *vs* 3
1) Before implant placement (n=16) 2) *Ex-post* value (after 5 years) 3) *Ex-post* value corrected	5.43 3.25 3.62	3.44-7.43 1.80-4.69 2.10-5.14	0.0365	0.070
1) 6 months after implant placement (n=15) 2) *Ex-post* value (after 5 years) 3) *Ex-post* value corrected	9.66 8.00 8.46	8.95-10.38 6.83-9.16 7.38-9.55	0.0167	0.0346
1) 24 months after implant placement (n=16) 2) *Ex-post* value (after 5 years) 3) *Ex-post* value corrected	9.68 8.81 9.25	9.26-10.11 8.02-9.59 8.65-9.84	0.0842	0.3216

**Table 2:** Willingness to pay (WTP) and willingness to accept (WTA) in Swiss Francs for two interforaminal implants in 16 edentulous patients.

Patient	WTP (CHF)	WTA (CHF)
1	1500	∞
2	1700	3000
3	3000	∞
4	3000	∞
5	3400	4500
6	3500	∞
7	4000	∞
8	4000	∞
9	4000	30000
10	4000	∞
11	4500	∞
12	5000	∞
13	5000	30000
14	10000	100000
15	12500	∞
16	-	∞
